# A Genomic Study of DNA Alteration Events Caused by Ionizing Radiation in Human Embryonic Stem Cells via Next-Generation Sequencing

**DOI:** 10.1155/2016/1346521

**Published:** 2015-11-22

**Authors:** Van Nguyen, Irina V. Panyutin, Igor G. Panyutin, Ronald D. Neumann

**Affiliations:** Nuclear Medicine Division, Department of Radiology and Imaging Sciences, Clinical Center, National Institutes of Health, 9000 Rockville Pike, Bethesda, MD 20892, USA

## Abstract

Ionizing radiation (IR) is a known mutagen that is widely employed for medical diagnostic and therapeutic purposes. To study the extent of genetic variations in DNA caused by IR, we used IR-sensitive human embryonic stem cells (hESCs). Four hESC cell lines, H1, H7, H9, and H14, were subjected to IR at 0.2 or 1 Gy dose and then maintained in culture for four days before being harvested for DNA isolation. Irradiation with 1 Gy dose resulted in significant cell death, ranging from 60% to 90% reduction in cell population. Since IR is often implicated as a risk for inducing cancer, a primer pool targeting genomic “hotspot” regions that are frequently mutated in human cancer genes was used to generate libraries from irradiated and control samples. Using a semiconductor-based next-generation sequencing approach, we were able to consistently sequence these samples with deep coverage for reliable data analysis. A possible rare nucleotide variant was identified in the *KIT* gene (chr4:55593481) exclusively in H1 hESCs irradiated with 1 Gy dose. More extensive further studies are warranted to assess the extent and distribution of genetic changes in hESCs after IR exposure.

## 1. Introduction

Human embryonic stem cells (hESCs) are derived from the inner cell mass of a blastocyst. They are capable of maintaining pluripotency under nondifferentiating culture condition and forming committed cell lineages that are the precursors of all three primary germ layers: ectoderm, endoderm, and mesoderm [1, 2]. The human fetus is known to be highly susceptible to genotoxic insults, possibly including but not limited to ionizing radiation (IR) [3]. hESCs were found to be highly radiosensitive and prone to apoptosis [4]. In culture, hESCs lack the G1/S checkpoint, with the majority of the cell population in the S phase constantly replicating their DNA [5, 6]. It was suggested that hESCs repair DNA damage induced by ultraviolet radiation via translesion synthesis, which makes them especially prone to mutagenesis [7].

Our laboratory uses hESC-based culture model to examine the biological effects of IR. Exposure to IR might cause DNA damage that could lead to mutation either during DNA replication or as a result of processing during DNA repair. The traditional approach to study IR-induced mutagenesis is based on selection and clonal expansion of the mutants. However, hESCs grow in colonies, and disruption of these colonies to single cells results in a very low plating efficiency of <1% [8]. Treatment with certain drugs, such as a ROCK (rho-associated, coiled-coil containing protein kinase) inhibitor, increases the plating efficiency but could compromise the cells' pluripotency [9]. Therefore, the clonal selection and expansion approach is not plausible for studying hESCs. In addition, expanding cells from single colonies would introduce a bias for the mutations that result in growth advantage in culture.

We hypothesized that radiation-induced primary genetic changes occur nonrandomly along a DNA molecule yet concentrate within “hotspot” regions of the genome, and, thus, they could be detected via deep sequencing. Depth of coverage is of significant importance in confidently detecting a DNA variant occurring in a small fraction of the tested cells. Whole human genome and even whole exome sequencing with coverage of more than 100x are still quite expensive at present. Since IR is often implicated as a risk for inducing cancer, we hypothesized that IR-induced changes in DNA sequence would most likely occur within cancer-related “hotspot” regions of the genome. Therefore, we focused on a portion of the human genome that includes multiple regions of cancer-related genes containing numerous mutation hotspots. In other words, we aimed to test whether the increased occurrence of IR-induced DNA changes contributed to the increased rate of mutation at these hotspots.

Next-generation sequencing (NGS) was first introduced commercially in 2005; it has since undergone dramatic improvements in read length, accuracy, coverage depth, and affordability [10]. In the field of cancer research, NGS is used for both cancer predisposition gene discovery and clinical validation, implying its tremendous potential in personalized medicine [11–14]. The work described in this study was conducted using the Ion Torrent Personal Genome Machine (Ion PGM, Life Technologies, Grand Island, NY, USA), which is one of the most popular NGS platforms. Its methodology is based on the detection of a hydrogen ion released alongside the pyrophosphate when DNA polymerase adds a dNTP to a strand of elongating DNA [15]. Using two independent data analysis software programs, we found one genetic alteration occurring exclusively in H1 hESC line irradiated with 1 Gy dose, suggesting that, in these highly sensitive hESCs, IR-induced genetic alteration events may be distributed nonrandomly within the DNA sequence.

## 2. Materials and Methods

### 2.1. Cell Culture and Irradiation

H1, H7, H9, and H14 hESCs (WiCell, Madison, WI, USA) were routinely cultured in mTeSR-1 medium (Stemcell Technologies, Vancouver, BC, Canada) on a BD Matrigel hESC-qualified basement membrane matrix (BD Biosciences, San Jose, CA, USA) at 37°C and 5% CO_2_ according to the manufacturers' protocols. Cells were subcultured every 5–7 days using collagenase IV (Invitrogen, Carlsbad, CA, USA) or dispase (Stemcell Technologies) as instructed by the manufacturers.

Cell cultures were exposed to 0.2 or 1 Gy of IR delivered by an Eldorado 8 ^60^Co teletherapy unit (MDS Nordion, Ottawa, ON, Canada, formerly Atomic Energy of Canada, Ltd.), then allowed to recover in a humidified CO_2_ incubator, and collected at four days after irradiation for analysis. In parallel, the control cell cultures were treated with sham-radiation.

For colony area measurement, hESCs were grown on gridded plates to help locate the colonies as previously described [16]. At various time points, phase-contrast pictures of individual colonies were taken using Zeiss Axiovert 200 microscope (Carl Zeiss, Thornwood, NY, USA), and the area of each colony was measured with AxioVision Software (Carl Zeiss) by manually outlining its borders. The measurement was performed for at least 30 individual colonies per plate.

### 2.2. Next-Generation Sequencing

Genomic DNA (gDNA) was isolated from harvested hESCs using a PureLink Genomic DNA Mini Kit (Invitrogen) and quantified by a NanoVue Plus spectrophotometer (GE Healthcare Life Sciences, Piscataway, NJ, USA) according to the manufacturers' instructions.

A total of 50 ng of each gDNA sample was subjected to library preparation using the Ion AmpliSeq Library Kit 2.0 (Life Technologies, Grand Island, NY, USA) as instructed by the manufacturer. The Ion AmpliSeq Cancer Hotspot Panel v2 (Life Technologies) consisting of 207 primer pairs was used to perform multiplex PCR for the generation of amplicon libraries from “hotspot” regions that are frequently mutated in human cancer genes. Such targets include approximately 2,800 mutations of 50 oncogenes and tumor suppressor genes described in the Catalogue of Somatic Mutations in Cancer (COSMIC). These genes are* ABL1*,* AKT1*,* ALK*,* APC*,* ATM*,* BRAF*,* CDH1*,* CDKN2A*,* CSF1A*,* CTNNB1*,* EGFR*,* ERBB2*,* ERBB4*,* EZH2*,* FBXW7*,* FGFR1*,* FGFR2*,* FGFR3*,* FLT3*,* GNA11*,* GNAS*,* GNAQ*,* HNF1A*,* HRAS*,* IDH1*,* IDH2*,* JAK2*,* JAK3*,* KDR*,* KIT*,* KRAS*,* MET*,* MLH1*,* MPL*,* NOTCH1*,* NPM1*,* NRAS*,* PDGFRA*,* PIK3CA*,* PTEN*,* PTPN11*,* RB1*,* RET*,* SMAD4*,* SMARCB1*,* SMO*,* SRC*,* STK11*,* TP53,* and* VHL*. Unique sequencing barcode adaptors from the Ion Xpress Barcode Adapters Kit (Life Technologies) were ligated to the amplicons to allow for sample multiplexing. All prepared libraries were quantified using Bioanalyzer high-sensitivity DNA chips (Agilent Technologies, Santa Clara, CA, USA) according to the manufacturer's protocol.

Each uniquely barcoded library was diluted in nuclease-free water to a stock concentration of 100 pM. The samples were then pooled and clonally amplified onto Ion Sphere particles (ISP, Life Technologies) by emulsion PCR according to the manufacturer's protocol for the Ion OneTouch 200 Template Kit v2 DL (Life Technologies) or the Ion PGM Template OT2 200 Kit (Life Technologies). A set of three libraries, including the control, low-dose (0.2 Gy), and high-dose (1 Gy) samples of the same cell line, was pooled for sequencing on an Ion 314 chip (Life Technologies), whereas two sets with a total of six libraries were pooled when an Ion 318 chip (Life Technologies) was used. Template-positive ISPs were then selectively isolated using the Ion Torrent OneTouch ES module (Life Technologies) as instructed by the manufacturer.

Semiconductor-based NGS was performed on an Ion PGM system (Life Technologies) using the Ion PGM Sequencing 200 Kit (Life Technologies) or Ion PGM Sequencing 200 Kit v2 (Life Technologies) according to the manufacturer's protocol. In the experiments to resequence the same DNA samples (Ion 318 chip-2, Table 3) we used Ion PGM Hi-Q Sequencing Kit. The successful sequencing of a sample set was determined based on the manufacturer's recommendations of expected throughput and total number of reads with a quality score of AQ20 (one misaligned base per 100 bases) for each chip type.

### 2.3. Data Analysis

#### 2.3.1. Ion Torrent Suite Software

Base calling and alignment of sample sequence to the reference genome (hg19) were performed by the Torrent Suite software version 4.0.2 (Life Technologies). All sequencing reads were automatically barcode sorted with low-quality reads removed. Identification of variants was achieved via the use of Ion Torrent Variant Caller Plugin version 4.0 (Life Technologies) with all parameters set as per the manufacturer's recommendation for the analysis of Ion AmpliSeq Cancer Hotspot Panel v2 data. Additional analysis included visual inspection of the read alignment and the presence of nucleotide variants on the Integrative Genomics Viewer (IGV, Broad Institute, Boston, MA) to confirm the variant calls by checking for possible sequencing errors.

#### 2.3.2. NextGENe Software

NextGENe software package version 2.3.4 (SoftGenetics, State College, PA) was also used for parallel analysis of sequencing data. For each sample set, the fastq output file was converted into a fasta file, and reads were aligned to the hg19 reference genome using default settings. Subsequently, identification of variants at frequency as low as 2% was performed with a minimum coverage of 400x for the reference sequence and 10x for the variant reads. Additionally, the number of forward (*F*) and reverse (*R*) reads had to be balanced with *F*/*R* ratio of >0.25 for a variant call to be accepted. Annotation by the software was confirmed by direct inspection of the aligned reads on the NextGENe Viewer. However, in the preliminary studies using Ion 314 chips, all variants with a minimum coverage of 100x and frequency as low as 1% were manually reviewed to avoid false-negative calls.

**Figure 1:**
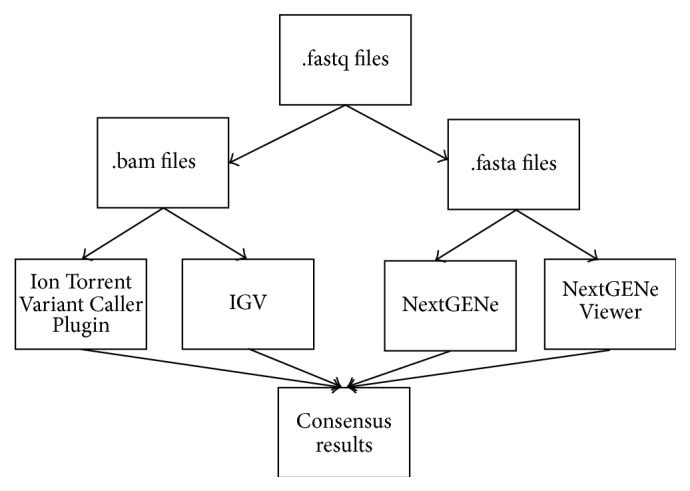
Analysis pipeline of sequencing data. Identification of variants was performed using two parallel analysis tools: Ion Torrent Variant Caller Plugin version 4.0 (Life Technologies) with visual inspection of the read alignment on the Integrative Genomics Viewer (IGV, Broad Institute, Boston, MA) and NextGENe software package version 2.3.4 (SoftGenetics, State College, PA) with the read alignment directly inspected on the NextGENe Viewer.

Results from both analysis platforms were compared, and consensus findings were reported for each data set (Figure 1).

## 3. Results

**Figure 2:**
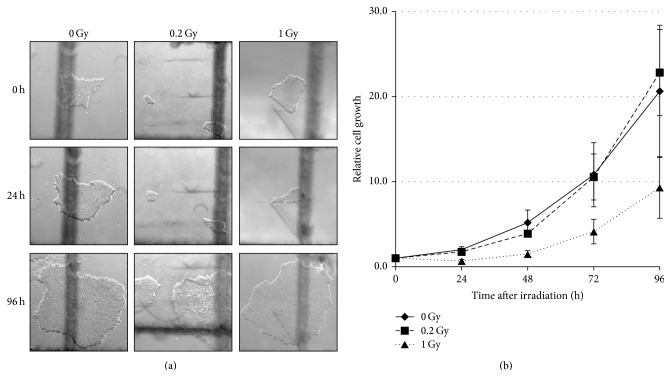
H14 human embryonic stem cell growth after exposure to ionizing radiation. (a) Microscopic images of representative colonies at 0, 24, and 96 hours after exposure to 0, 0.2, or 1 Gy of ionizing radiation. (b) Cell growth curves of H14 human embryonic stem cells irradiated with 0, 0.2, or 1.0 Gy. The area of all colonies was normalized to 1 at the 0-hour time point. Averages of the measurement of 20 colonies are presented; error bars represent standard deviation.

As we previously described and commonly observed, irradiation at 1 Gy readily killed hESCs with a large quantity of the cells undergoing apoptosis within 24 hours after exposure [17, 18]. The massive cell death was clearly observable via the presence of floating debris and “holes” within cell colonies (Figure 2(a)). Because hESCs only grow in colonies and most of them die if a colony is broken down to individual cells, we developed a method of assessing their growth curves based on the measurement of the colony area [16].  Figure 2(b) shows such growth curves measured for H14 hESCs line as an example. Forty-eight hours after exposure to 1 Gy, most of the colonies recovered and continued to grow at the same rate as the control cells. Similar growth curves were obtained for the other three hESCs lines under investigation. The percentage of cells remaining in colonies at 24 hours after 1 Gy IR as compared to the sham-irradiated controls varied from 10% to 40% for these lines, with H1 cells being the most radiosensitive (Table 1). These results demonstrate that 1 Gy irradiation led to significant cell death; however, exposure to 0.2 Gy caused just a slight delay in the growth curves as shown in Figure 2(b) for the H14 hESCs line and Table 1. Nonetheless, cells that survived and successfully repopulated after four days in culture were harvested for sequencing experiments.

In the preliminary pilot studies, we used Ion 314 chips to sequence a set of three libraries (1 Gy, 0.2 Gy, and 0 Gy control) pooled from the same cell line in each run. This design allowed us to minimize the effects of technical variation between sequencing runs and to directly compare the results from each sample set. All sequencing experiments with Ion 314 chips met or exceeded the technical requirements of the manufacturer for successful runs. We were able to sequence all samples at adequate coverage for data analysis according to the manufacturer's guidelines. The mean depth coverage for each sample varied from 233.4x to 830.5x (Table 1). Afterwards, the sequencing was repeated with higher capacity Ion 318 chips. In this case, the 1 Gy, 0.2 Gy, and 0 Gy control samples of two cells lines (for a total of six samples) were pulled on the same chip. The mean depth coverage for the samples on the Ion 318 chips varied from 2,135x to 3,934x, which are five to 10 times higher than those of the Ion 314 chips (Table 2). Next, we repeated the experiment by preparing another set of libraries from the same DNA samples and subjected them to sequencing on Ion 318 chips (318 chip-2, Table 3). Detection of variants was performed by two independent software platforms, Ion Torrent Variant Caller Plugin version 4.0 (Life Technologies) and NextGENe software package version 2.3.4 (SoftGenetics).

The majority of variants were present in both irradiated and control cells and were either heterozygous (≈50% frequency) or homozygous (≈100% frequency), reflecting the genetic differences (or single nucleotide polymorphism) between hESC lines and the reference genome, but not genomic changes resulting from IR exposure. These variants are listed in Supplemental Table 1 (see Supplementary Material available online at http://dx.doi.org/10.1155/2016/1346521). However, a few rare variants were detected only in the samples irradiated with 1 Gy IR, but not in the control samples or in those irradiated with 0.2 Gy IR. These rare variants are presented in Table 3. Three of the six rare variants detected in the experiments with 314 chips were not confirmed by the 318 chip results, due to either low coverage or unreliable frequency.

**Figure 3:**
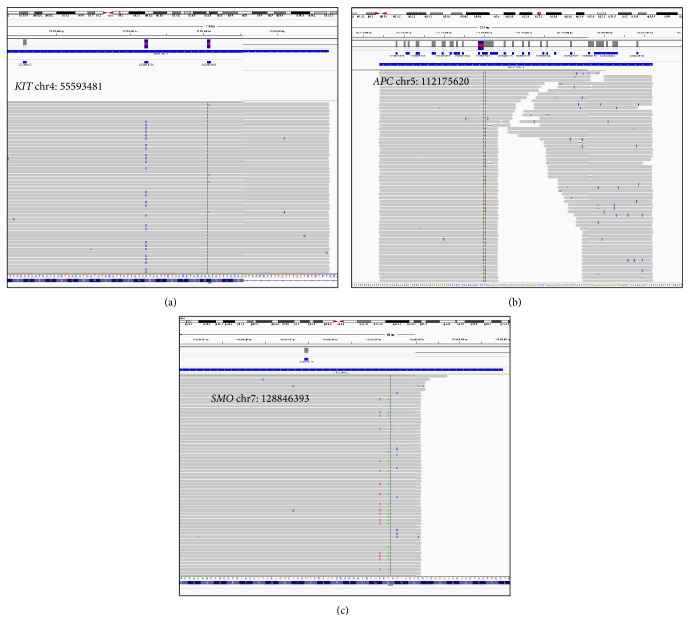
Representative snapshots of Integrative Genomics Viewer (IGV, Broad Institute, Boston, MA) showing the aligned reads for the rare variants detected in samples exposed to 1 Gy of ionizing radiation. (a) At position chr4:55593481 in the* KIT* protooncogene of H1 cells. (b) At position chr5:112175620 in the tumor suppressor gene* APC* of H9 cells. (c) At position chr7:128846393 in the* SMO *oncogene of H9 cells. Each horizontal grey bar represents an individual read with the variant highlighted with vertical columns (A > G in panel (a), and T > G in panel (b), and T > A in panel (c)).

The only rare variant detected in all three experiments using 314 and 318 chips is located in the “hotspot” regions of cancer-related gene* KIT* (Table 2). In the* KIT* gene of H1 hESCs exposed to 1 Gy, an A-to-G variation at the position chr4:55593481 was detected at a frequency of 2.7% (314 chip), 3.1% (318 chip), or 3.1% (318 chip-2). Two variants were detected in H9 hESCs exposed to 1 Gy IR in two out of three sequencing experiments as follows: a T-to-G variation in the* APC* gene at position chr5:112175620 with frequencies of 7.4% (314 chip) and 8.2% (318 chip) and a T-to-A variation in the* SMO* gene at position chr7:128846393 with frequencies of 2.22% (314 chip) and 3.5% (318 chip). However, while the variant in the* KIT* gene (H1 hESCs) appeared in the normal full-length reads covering whole amplicons (Figure 3(a)), those in the* APC* and* SMO* genes occurred almost exclusively close to the ends of incompletely aligned reads that covered only a portion of the corresponding amplicons (Figures 3(b) and 3(c)). The T-to-A variant in the* SMO* gene at position chr7:128846393 also closely correlated with the adjacent C-to-T variant located two nucleotides upstream at chr7:128846391 (Figure 3(c)). In addition, the two variants called in H9 hESCs were not confirmed after resequencing on the same DNA samples (column “318 chip-2,” Table 2). These resequencing runs utilized Hi-Q polymerase (see Materials and Methods), which yielded considerably less incompletely aligned reads. We did not find any rare variants that were present in the control samples or in the samples irradiated with 0.2 Gy dose but not in the 1 Gy irradiated samples.

Taken together, our results suggest that the higher dose of IR (1 Gy) exerted acute toxic effects on hESCs, resulting in significant cell death after exposure. It also produced at least one rare variant within the cancer “hotspot” regions of the surviving cells.

## 4. Discussion

In the present study, we investigated the biological effects of IR on four different hESC lines using a deep sequencing approach. Cells were subjected to IR at low (0.2 Gy) or high (1 Gy) dose and then maintained in culture for four days to allow for repair of DNA damage before being harvested for DNA isolation. As IR is often implicated as a risk for cancer induction, a primer pool targeting genomic “hotspot” regions that are frequently mutated in human cancer genes was used to generate libraries from low- and high-dose and control hESC samples. These samples were then subjected to semiconductor-based targeted deep sequencing.

It was estimated that exposure to 1 Gy of IR results in approximately 4,000–5,000 DNA damage incidents per cell [19]. Because the cancer “hotspot” libraries generated in this study typically included 207 amplicons with an average size of 150 base pairs each, they would thus cover a very small portion (≈0.001%) of the human genome. Therefore, if DNA damage was distributed uniformly along the genome, the number of such damage incidents within the regions of interest would be minimal. However, the genomic areas included in this panel are well known to play critical roles in carcinogenesis, with genes frequently mutated in various malignancies such as* APC* in colon cancer [20],* BRAF* in melanoma or hairy cell leukemia [21],* EGFR* in non-small cell lung cancer, and* IDH1* in glioblastoma multiforme [22] to name a few. It is worthy of note that the panel also included genes reportedly associated with the types of cancer that have been linked to radiation exposure by epidemiological studies such as leukemia and brain tumors [23]. Furthermore, as most DNA damage incidents from IR exposure are effectively repaired [19] and only misrepaired and/or nonrepaired damage could potentially cause mutations and participate in the carcinogenesis [24], these “hotspot” regions might either be more susceptible to radiation-induced damage or involve damage that is more significant for cancer development. In addition, currently it is not technically possible to sequence the entire human genome at the coverage achievable with targeted sequencing in order to identify extremely rare and subtle genomic changes. Therefore, our experimental approach was to use the highly sensitive hESCs as our model and to focus on genomic regions that are known to be crucial for cancer development. Moreover, NGS has been successfully utilized to detect somatic mutations within various cancer genes, with reported detection limits frequently in the range of 5–10% to as low as 1-2% [13, 25, 26]. Thus, it is expected that rare variants that resulted from IR exposure should be detectable at our coverage exceeding 1,000x.

Most of the detected variants were present in both irradiated and control samples (Supplemental Table 1) and had either ≈100% frequency (homozygous) or ≈50% frequency (heterozygous), indicating intrinsic differences between the tested genomes versus reference rather than changes occurring as a result of radiation exposure. The rare variant in the* KIT* gene that was detected only in the H1 cell line after 1 Gy irradiation is a silent mutation (K546K) and thus unlikely to provide a growth advantage to the cells. This variation is listed in the COSMIC database under reference number COSM21983 and was found in cancers of the large intestine, bone, and soft tissues.

Interestingly, we did not detect variant in the* KIT* gene in other hESC lines, nor when we sequenced another library obtained from a different round of hESC irradiation (data not shown). This may reflect the highly stochastic nature of the IR-induced damage distribution along cellular DNA. The fact that in sequencing the same DNA and library we consistently detected this variant indicates that it occurred in multiple cells; and the mere existence of this variant implies that distribution of DNA variants after IR exposure could be nonrandom. We recognize that detection of just one variant is not statistically significant and could be explained by other unforeseen reasons, for example, by cross-contamination. We believe that to assess the degree of nonuniformity of the variant distribution after IR exposure, sequencing larger portions of the genome is required. Although highly unlikely due to the short (four days) time in culture after irradiation, the selective expansion of low-frequency preexisting variants in these particular cells could have contributed to the frequency of the* KIT* gene variant; a deeper sequencing of the regions containing the variants in question in future experiments could address such an issue.

In conclusion, our results from this genomic study suggest that exposure to lower dose of IR (0.2 Gy) did not result in a detectable increase in genetic alteration events occurring within multiple cancer “hotspot” regions of the genome in the highly sensitive hESCs. Nonetheless, the higher dose of IR (1 Gy) led to massive cell death within 24 hours after exposure and resulted in one possible variation in one of the four hESC lines tested. We believe that our research paves the way for further studies of genetic alterations in human cells caused by IR using NGS. With the development of innovative, more precise, and economical NGS strategies, it should be possible to sequence increasingly larger portions of the genome with progressively deeper coverage. Future studies using more sophisticated technologies are warranted to provide direct experimental evidence for the distribution of the genetic variants after exposure to IR.

## Supplementary Material

Supplementary Table 1 lists variants that were present in both irradiated and control cells and were either heterozygous (≈50% frequency) or homozygous (≈50% frequency) reflecting the genetic differences (or single nucleotide polymorphism) between the hESC lines tested and reference, but not genomic changes resulted from IR exposure. Identification of all variants was performed using two parallel analysis tools, Ion Torrent Variant Caller plugin version 4.0 (Life Technologies) and NextGENe software version 2.3.4 (SoftGenetics), with appropriate visual inspection of all alignments.

## Figures and Tables

**Table 1:** Percent of cells which survived at 24 hours after IR exposure relative to the unexposed control.

	H1	H7	H9	H14
0.2 Gy	49	78	83	87
1.0 Gy	11	20	31	34

**Table 2:** Mean depth coverage.

Sample name	Ion 314 chip	Ion 318 chip
H1 0 Gy	830.5	3,683
H1 0.2 Gy	573.2	3,687
H1 1 Gy	659.0	3,934
H7 0 Gy	540.1	3,556
H7 0.2 Gy	463.2	3,702
H7 1 Gy	547.8	3,799
H9 0 Gy	545.2	3,238
H9 0.2 Gy	535.8	3,174
H9 1 Gy	233.4	2,135
H14 0 Gy	800.4	3,550
H14 0.2 Gy	631.9	3,235
H14 1 Gy	633.8	3,492

**Table 3:** Rare variants identified in the four human embryonic stem cell lines after exposure to 1 Gy of ionizing radiation.

Cell line	Position	Gene	Reference	Variant	314 chip		318 chip		318 chip-2	
					Frequency	Coverage	Frequency	Coverage	Frequency	Coverage
H1	chr4:55593481	*KIT *	A	G	2.7%	673	3.1%	4,325	3.1%	4,870

H7	chr11:108225561	*ATM *	T	C	1.25%	320	—	2,486	—	1,407

H9	chr5:112175620	*APC *	T	G	7.4%	269	8.2%	1,798	—	3,089
	chr7:55249049	*EGFR *	A	G	5.5%	109	—	994	—	1,416
	chr7:128846391	*SMO *	C	T	1.25%	319	—	3121	—	3,524
	chr7:128846393	*SMO *	T	A	2.22%	316	3.5%	3,228	—	3,526
