# Supplementary material for: A Genomic Study of DNA Alteration Events Caused by Ionizing Radiation in Human Embryonic Stem Cells via Next-Generation Sequencing
Source: Stem Cells Int. 2015 Nov 22;2016:1346521. doi: 10.1155/2016/1346521 (PMC4670683; doi:10.1155/2016/1346521)
Supplement: Supplementary file 1 — Supplementary Table 1 lists variants that were present in both irradiated and control cells and were either heterozygous (≈50% frequency) or homozygous (≈50% frequency) reflecting the genetic differences (or single nucleotide polymorphism) between the hESC lines tested and reference, but not genomic changes resulted from IR exposure. Identification of all variants was performed using two parallel analysis tools, Ion Torrent Variant Caller plugin version 4.0 (Life Technologies) and NextGENe software version 2.3.4 (SoftGenetics), with appropriate visual inspection of all alignments. [file 1346521.f1.pdf]

Supplemental Table 1. Variants detected in the four human embryonic stem cell lines that were not related to ionizing radiation exposure

Cell line	Position	Gene	Reference	Variant	Frequency on 314™ chip			Frequency on 318™ chip		
					Control	0.2 Gy	1 Gy	Control	0.2 Gy	1 Gy
H1	chr4:1807894	FGFR3	G	A	100%	100%	100%	100%	100%	100%
	chr4:55141055	PDGFRA	A	G	100%	99.8%	100%	100%	100%	100%
	chr4:55593464	KIT	A	C	47.3%	47.3%	50.4%	46.6%	48%	47.4%
	chr5:112175770	APC	G	A	99.8%	100%	100%	99.9%	100%	100%
	chr7:55249063	EGFR	G	A	56.1%	48.7%	53.2%	52.8%	51%	51.3%
	chr10:43613843	RET	G	T	100%	100%	98.6%	100%	100%	100%
	chr22:24176287	SMARCB1	G	A	52.7%	50.3%	46.8%	52.8%	50.1%	48.8%
H7	chr4:1807894	FGFR3	G	A	100%	100%	99.7%	100%	100%	100%

	chr4:55141055	PDGFRA	A	G	86%	96.5%	69.1%	100%	100%	100%
	chr4:55593481	KIT	A	G	100%	100%	100%	100%	100%	100%
	chr4:55946171	KDR	G	A	51.9%	46.0%	51.6%	52.7%	49.7%	49.7%
	chr4:55972974	KDR	T	A	50.7%	52.2%	44.0%	48.4%	49.3%	50.6%
	chr5:112175770	APC	G	A	100%	100%	99.1%	100%	100%	100%
	chr7:55249063	EGFR	G	A	100%	100%	100%	100%	100%	100%
	chr10:43613843	RET	G	T	51.4%	46.9%	53.6%	53.9%	51.8%	52.4%
	chr10:43615633	RET	C	G	45.1%	49.6%	50.3%	49.3%	47.9%	51.6%
	chr19:1220321	STK11	T	C	54.1%	50.4%	50.4%	52%	54.7%	51.3%
	chr22:24176287	SMARCB1	G	A	51.6%	52.2%	45.6%	50.4%	51.6%	49.1%
H9	chr2:212812097	ERBB4	T	C	66.9%	61.0%	62.4%	66.2%	60.1%	59.6%
	chr3:178917005	PIK3CA	A	G	99.8%	100%	97.7%	98.9%	100%	98.7%

	chr4:1807894	FGFR3	G	A	100%	100%	99.7%	100%	100%	100%
	chr4:55141055	PDGFRA	A	G	99.8%	100%	98.3%	100%	100%	100%
	chr5:112175770	APC	G	A	98.9%	99.1%	98.9%	100%	100%	100%
	chr7:55249063	EGFR	G	A	52.7%	46.2%	54.8%	50.4%	51.1%	49.9%
	chr10:43613843	RET	G	T	100%	100%	100%	100%	100%	100%
	chr19:1220321	STK11	T	C	49.6%	54.5%	43.7%	53.4%	56.1%	49.4%
H14	chr2:29432625	ALK	C	A	21%	32.6%	37.6%	27.3%	32.2%	38.3%
	chr4:1807894	FGFR3	G	A	100%	99.6%	100%	100%	100%	100%
	chr4:55141055	PDGFRA	A	G	100%	100%	99.8%	100%	100%	100%
	chr4:55593481	KIT	A	G	100%	100%	100%	100%	100%	100%
	chr4:55946171	KDR	G	A	49.9%	47%	49.9%	48.6%	50.8%	50.7%
	chr4:55972974	KDR	T	A	51.6%	50.8%	50.8%	50.6%	50.6%	51.6%

	chr5:112175770	APC	G	A	100%	100%	100%	100%	100%	100%
	chr7:55249049	EGFR	A	G	100%	100%	100%	100%	100%	100%
	chr19:1220321	STK11	T	C	100%	100%	100%	100%	100%	100%
	chr22:24176287	SMARCB1	G	A	52.6%	48.9%	48.0%	51%	50.2%	48%
